# The Association Between State-Level Racial Attitudes Assessed From Twitter Data and Adverse Birth Outcomes: Observational Study

**DOI:** 10.2196/17103

**Published:** 2020-07-06

**Authors:** Thu T Nguyen, Nikki Adams, Dina Huang, M Maria Glymour, Amani M Allen, Quynh C Nguyen

**Affiliations:** 1 Department of Family and Community Medicine University of California, San Francisco San Francisco, CA United States; 2 Applied Research Laboratory for Intelligence and Security University of Maryland College Park, MD United States; 3 Department of Epidemiology and Biostatistics University of Maryland School of Public Health College Park, MD United States; 4 Department of Epidemiology and Biostatistics University of California, San Francisco San Francisco, CA United States; 5 Divisions of Community Health Sciences and Epidemiology University of California, Berkeley Berkeley, CA United States

**Keywords:** social media, racial bias, birth outcomes, racial or ethnic minorities

## Abstract

**Background:**

In the United States, racial disparities in birth outcomes persist and have been widening. Interpersonal and structural racism are leading explanations for the continuing racial disparities in birth outcomes, but research to confirm the role of racism and evaluate trends in the impact of racism on health outcomes has been hampered by the challenge of measuring racism. Most research on discrimination relies on self-reported experiences of discrimination, and few studies have examined racial attitudes and bias at the US national level.

**Objective:**

This study aimed to investigate the associations between state-level Twitter-derived sentiments related to racial or ethnic minorities and birth outcomes.

**Methods:**

We utilized Twitter’s Streaming application programming interface to collect 26,027,740 tweets from June 2015 to December 2017, containing at least one race-related term. Sentiment analysis was performed using support vector machine, a supervised machine learning model. We constructed overall indicators of sentiment toward minorities and sentiment toward race-specific groups. For each year, state-level Twitter-derived sentiment data were merged with birth data for that year. The study participants were women who had singleton births with no congenital abnormalities from 2015 to 2017 and for whom data were available on gestational age (n=9,988,030) or birth weight (n=9,985,402). The main outcomes were low birth weight (birth weight ≤2499 g) and preterm birth (gestational age <37 weeks). We estimated the incidence ratios controlling for individual-level maternal characteristics (sociodemographics, prenatal care, and health behaviors) and state-level demographics, using log binomial regression models.

**Results:**

The accuracy for identifying negative sentiments on comparing the machine learning model to manually labeled tweets was 91%. Mothers living in states in the highest tertile for negative sentiment tweets referencing racial or ethnic minorities had greater incidences of low birth weight (8% greater, 95% CI 4%-13%) and preterm birth (8% greater, 95% CI 0%-14%) compared with mothers living in states in the lowest tertile. More negative tweets referencing minorities were associated with adverse birth outcomes in the total population, including non-Hispanic white people and racial or ethnic minorities. In stratified subgroup analyses, more negative tweets referencing specific racial or ethnic minority groups (black people, Middle Eastern people, and Muslims) were associated with poor birth outcomes for black people and minorities.

**Conclusions:**

A negative social context related to race was associated with poor birth outcomes for racial or ethnic minorities, as well as non-Hispanic white people.

## Introduction

Preterm birth and low birth weight (LBW) are the leading causes of infant mortality and childhood disability [1,2]. In the United States, racial disparities in birth outcomes persist [3,4] and have been widening [5]. In 2017, the preterm birth rate was 9.05% for non-Hispanic white mothers but 13.93% for black mothers. The LBW rate among black infants has consistently been more than twice that among non-Hispanic white infants from 2006 to 2016 [6]. Maternal health behaviors, adequacy of prenatal care, and sociodemographic characteristics do not fully explain the observed disparities [3]. There is increasing evidence that racial bias may partially contribute to these persistent disparities [3,7,8].

Traditionally, experiences with discrimination are assessed at the individual level by self-reports [9,10]. Self-reported racial attitudes and beliefs are subject to a number of limitations including social desirability bias and self-censorship [11,12], risking invalid exposure assessment [13,14]. Self-reports of racial discrimination can be influenced by a variety of factors including coping (eg, denial), trait- or state-based aspects of personality (eg, stigma consciousness and race-based rejection sensitivity), and aspects of racial identity (eg, internalized racism) [13]. While individual self-reported experiences of discrimination can provide important information, the social climate of a place represents a complimentary aspect of racial bias and discrimination that may have its own influence on health, independent of individual-level experiences. Thus, relying only on individual self-reported data can underestimate the effect of racism on health.

There are several mechanisms by which discrimination may impact poor birth outcomes. For example, the experience of discrimination may activate a stress response that may contribute to poor birth outcomes if experienced chronically. Maternal stress may impact birth outcomes through the following three major pathways: (1) altered neuroendocrine function, which leads to activation of the maternal-placental-fetal endocrine system that promotes childbirth [15,16]; (2) altered immune function that results in increased susceptibility to infections and inflammatory responses [17]; and (3) maladaptive coping behaviors, such as smoking and alcohol consumption [18]. Discrimination is also hypothesized to influence birth outcomes through access to resources, such as education, employment, health care, and housing [3], but these are long-term processes.

An innovative study highlighted the potential impact of a race- or ethnicity-related event that creates a change in the contextual-level social climate. The authors investigated birth outcomes after a federal immigration raid in Postville, Iowa in 2008, which at the time, was the largest single-site raid in US history [19]. Comparing the birth weight of infants born in the 37 weeks after the raid in Iowa with the same 37-week period 1 year prior, Latina mothers, including US-born Latina mothers, experienced a 24% increase in the risk of having an LBW infant after the raid. Changes in LBW were not observed for non-Latina white mothers. The investigators conducted a state-level analysis and found estimated effects in not only Postville but also the state of Iowa. Another study found that Arab-named women experienced a relevant increase in the risk of having an LBW or preterm infant following the September 11, 2001, attacks on comparing the 6 months after the attacks to the same 6-month period 1 year prior [20]. These studies provide evidence for the potential influence of the social context on the health of affected communities.

Social media represents an under-used source of data for public health research. Millions of tweets are sent daily, and 90% of Twitter users have made their profile public [21]. In the web-based space, people express a variety of views and beliefs, including those that are related to race. In addition, research suggests that the sense of anonymity provided by web-based spaces emboldens people to express views they may not state during in-person interactions [22]. These aspects make social media an attractive source for capturing sensitive topics such as race-related discussions.

Previous studies have used Twitter data to examine topics, such as vaccination [23] and national patterns in nutrition, exercise, and happiness [24], and to conduct health surveillance [25]. However, little research has been performed to investigate sensitive topics, such as race and racism on social media, and previous studies examining racism using social media data have focused on hate speech [26] and racial slurs [27].

To provide a race- or ethnicity-related measure of the social climate and address prior limitations of self-reported individual-level measures, we developed a novel area-level measure of racial sentiment and examined its association with LBW and preterm birth. We took a broad approach and collected tweets referencing racial or ethnic groups, not just hate speech tweets or tweets using racial slurs. However, terms conventionally perceived as racial slurs can be used in nonderogatory ways, and such reappropriation is common on Twitter. For instance, in popular culture, the term “nigga” is often used as an in-group term without valuation [27]. Furthermore, discussions conveying racial sentiment can occur without the use of racial slurs. A more comprehensive examination of tweets using race-related terms may include a sentiment analysis of tweets using racial slurs, as well as neutral racial terms such as “black,” “African American,” or “Asian.” In a previous paper, we examined the association between racial sentiment derived from Twitter data and adverse birth outcomes in 2015 [28]. In this paper, we improve upon the accuracy of the machine learning model to label the sentiment of tweets, increase the sample size of tweets by 20 fold, and examine the relationships using Twitter and birth outcome data for multiple years rather than a single year.

## Methods

### Twitter Data

A random 1% sample of publicly available tweets was collected from June 2015 to December 2017, using Twitter’s Streaming application programming interface. The analysis included English language tweets from the United States with latitude and longitude coordinates or other “place” attributes that permitted the identification of the state where the tweet was associated. All tweets included in the sample also used one or more of the 518 identified race-related keywords (Multimedia Appendix 1). The terms were compiled from racial and ethnic categories used by the US census, prior studies examining race-related online conversations [27,29], and an online database of racial slurs [30]. Tweets were classified into the following five main racial or ethnic categories according to the keywords used: black, Hispanic, Asian, white, and Middle Eastern. The Middle Eastern category included tweets that were anti-Islamic or related to Muslims.

The Twitter data were cleaned and processed for the analysis. We removed duplicate tweets according to the “tweet_id.” We identified exclusion terms that tended to retrieve irrelevant tweets such as “black smoke” and “Indian Rd.” To prevent undue influence from a small number of very frequent users, we excluded tweets from users who tweeted more than 1000 times a year in the data set, which represented 3% to 4% of all tweets. In total, we collected 26,027,740 tweets from 2,498,717 Twitter users. This study was determined to be exempt by the Institutional Review Board of the University of California, San Francisco.

### Sentiment Analysis

We utilized support vector machine (SVM), a supervised machine learning model, to label the tweets. We obtained training data from manually labeled Sentiment140 (n=498) [31], Kaggle (n=7086) [32], and Sanders (n=5113) [33] and 6481 tweets labeled by our research group. Sentiment140, Kaggle, and Sanders datasets are publicly available training datasets specifically labelled for sentiment analysis. For our primary analysis, we compared negative tweets (assigned a value of 1) to all other tweets, which were positive or neutral tweets (assigned a value of 0). We used five-fold cross validation to assess the model performance and reached a high level of accuracy for the negative classification (91%) and a high F1 score (84%). Tweets were also labeled as positive or not positive. We similarly used five-fold cross validation and achieved an accuracy of 89% and a F1 score of 81%. State-level sentiment variables were created by averaging the dichotomous sentiment of tweets referencing various racial or ethnic groups.

### Individual-Level Health Data

We used data from the 2015-2017 restricted US natality files with geographic identifiers as individual-level birth outcome data. The files were obtained after submitting a research proposal to and obtaining approval for data access from the National Center for Health Statistics [34]. The analysis was restricted to singleton births with no congenital abnormalities. Congenital abnormalities [35] and twins, triplets, and other higher order multiple births increase the risk for LBW and preterm birth [36]. The primary outcomes were LBW (defined as birth weight ≤2499 g) and preterm birth (defined as gestational age <37 weeks). Models for preterm birth included data from 9,988,030 births and models for LBW included 9,985,402 births.

### Covariates

We adjusted for potential confounders of the association between racial sentiment and birth outcomes. Individual-level maternal characteristics included birth year, maternal age (linear spline with knots at 19, 25, 29, 33, and 38 years), race (white, non-Hispanic; black, non-Hispanic; American Indian/Alaskan Native, non-Hispanic; Asian, non-Hispanic; Native Hawaiian/Pacific Islander, non-Hispanic; multiracial, non-Hispanic), Hispanic ethnicity, marital status (married/unmarried), education (less than high school, high school or General Education Development [GED], some college, bachelor’s degree, master’s degree, or doctorate), body mass index (kg/m^2^), smoking during pregnancy (first, second, or third trimester), first birth (yes/no), and prenatal care initiation during the first trimester (yes/no). We also adjusted for state-level characteristics including proportions of non-Hispanic black and Hispanic individuals, population density (per square mile), southern state indicator (yes/no), and economic disadvantage (standardized factor score [37,38] summarizing the following variables [%]: unemployed; some college education, high school diploma, children in poverty, single parent household, and median household income) to account for state-level compositional differences in demographic and economic characteristics. Use of the factor score has been previously published [24]. State-level covariates were derived from 2013 to 2017 through 5-year estimates from the American Community Survey [39].

### Statistical Analysis

For each year, state-level sentiment toward racial or ethnic minorities was merged with data on births during that year. We estimated incidence ratios (IRs) using log binomial regression models, controlling for individual-level maternal characteristics and state-level demographic characteristics. In our main analyses, we modeled negative sentiment of tweets using race-related terms, but in the sensitivity analysis, we modeled the ratio of negative to positive sentiments to examine whether the results were robust for modeling different polarities of sentiment. We evaluated statistical significance at *P*<.05. Stata MP 15 (StataCorp LP, College Station, Texas, USA) was used for statistical analyses, and R software (R Foundation for Statistical Computing, Vienna, Austria) was used for mapping [40].

## Results

From 2015 to 2017, we collected 26,027,740 tweets containing at least one of the relevant keywords pertaining to a racial or ethnic group. Among the 518 terms assessed, 20 terms were present in 75% of all tweets with reference to a racial or ethnic minority group. The top Twitter terms were “nigga/niggas” (13,561,626/ 26,027,740, 52.10%), “racist” (1,070,770/ 26,027,740, 4.11%), “Mexican” (620,957/ 26,027,740, 2.39%), “white people” (514,111/ 26,027,740, 1.98%), and “Chinese” (498,775/ 26,027,740, 1.92%) (Table 1). Additionally, there were 15,683,909 tweets about black people, 1,801,780 about Asian people, 1,577,568 about white people, 1,512,566 about Hispanic people, and 1,274,827 about Middle Eastern people (Table 2). We have previously examined the emerging themes of tweets using race-related keywords [41]. Briefly, for negative sentiment tweets, tweets ranged from complaints about hassles in daily life (eg, “I hate when ppl Try to Join a Sport all late like niggah you didn't put in the work I did”) to race-related insults using derogatory language (eg, “Middle Eastern/Arabic accents piss me off more than most things”) and rare tweets expressing hostility or mentioning violence (eg, “if they are carrying a Mexican flag in Az. they need to be arrested.”) The use of “nigga” was common in negative sentiment tweets. However, Twitter users frequently use this term casually as slang.

**Table 1:** Top Twitter terms.

Term	Tweets (N=26,027,740), n (%)
Nigga	8,300,511 (31.89)
Niggas	5,261,115 (20.21)
Racist	1,070,770 (4.11)
Mexican	620,957 (2.39)
White people	514,111 (1.98)
Chinese	498,775 (1.92)
Racism	422,279 (1.62)
Muslim	381,601 (1.47)
Asian	312,520 (1.20)
Muslims	259,998 (1.00)
Japanese	238,588 (0.92)
Immigration	214,416 (0.82)
Indian	193,782 (0.74)
Islam	189,739 (0.73)
Syria	181,771 (0.70)
White girl	180,426 (0.69)
Jewish	170,040 (0.65)
Ghetto	167,128 (0.64)
Refugees	165,674 (0.64)
Black people	163,062 (0.63)

The geographic distributions of negative and positive sentiment tweets are displayed in Multimedia Appendix 2 and Multimedia Appendix 3, respectively. There was clustering of a higher proportion of negative tweets in the southeastern region of the United States (Multimedia Appendix 2) and clustering of a higher proportion of positive tweets in the western region of the United States (Multimedia Appendix 3). Twitter-derived measures of racial sentiment are presented in Table 2. Approximately 40.33% (9,657,039/23,945,052) of the tweets using race-related terms were categorized as negative. Tweets related to Middle Eastern people had the highest proportion of negative sentiment (638,688/1,274,827, 50.10%), whereas tweets related to Asian people had the lowest proportion of negative sentiment (113,172/1,801,780, 6.28%). Demographic characteristics of mothers giving birth from 2015 to 2017 are presented in Table 3. The mean age of mothers was 29 years, 59.74% (6,466,521/10,824,077) were married, and 85.99% (9,578,803/11,139,992) completed at least high school. Additionally, 6.37% (717,541/11,272,819) of singleton babies with no congenital abnormalities were born LBW and 7.91% (891,628/11,273,872) were born preterm.

**Table 2:** Negative sentiment for race-related terms used in tweets.

Race-related term	Number of tweets	Number of tweets with negative sentiment (%)
Racial or ethnic minorities	23,945,052	9,657,039 (40.33)
Black people	15,683,909	7,073,443 (45.10)
Middle Eastern people	1,274,827	638,688 (50.10)
Hispanic people	1,512,566	172,433 (11.40)
Asian people	1,801,780	113,172 (6.28)
White people	1,577,568	700,440 (44.40)

**Table 3:** Characteristics of mothers giving birth from 2015 to 2017.

Characteristic		Mean (SD) or n/N (%)	
Age, years		28.6 (5.82)	
Married		6,466,521/10,824,077 (59.74)	
White, non-Hispanic		5,852,869/11,187,000 (52.32)	
Black, non-Hispanic		1,600,020/11,187,000 (14.30)	
Asian, non-Hispanic		717,706/11,187,000 (6.42)	
Hispanic ethnicity		2,666,823/11,187,000 (23.84)	
US born		8,645,413/11,257,974 (76.79)	
**Education**			
	Less than high school		1,561,190/11,139,992 (14.01)
	High school		2,829,005/11,139,992 (25.40)
	Some college		3,238,463/11,139,992 (29.07)
	College		2,221,480/11,139,992 (19.94)
	Master’s or doctorate		1,289,855/11,139,992 (11.58)
**Birth outcomes**			
	Low birth weight		717,541/11,272,819 (6.37)
	Preterm birth		891,628/11,273,872 (7.91)

State-level racial sentiment was associated with LBW and preterm birth. In the entire population, mothers living in states with the highest level (third tertile) of negative tweets referencing racial or ethnic minorities had a 8% greater incidence of LBW (95% CI 1.04-1.13) and 8% greater incidence of preterm birth (95% CI 1.00-1.14) compared with mothers living in states with the lowest level (first tertile) of negative sentiment (Table 4). On investigating birth outcomes for racial or ethnic minorities, the direction and magnitude of effects were similar, with more negative tweets referencing racial or ethnic minorities being associated with a 13% increase in LBW (95% CI 1.06-1.21) and 10% increase in preterm birth (95% CI 1.05-1.16) among racial or ethnic minority mothers.

Examining sentiment toward specific groups, we found that states in the highest level (third tertile) of negative tweets referencing Middle Eastern people were associated with a greater incidence of LBW among racial or ethnic minorities (IR 1.07, 95% CI 1.02-1.12). More negative tweets referencing black people (IR 1.08, 95% CI 1.03-1.14) were associated with a greater incidence of LBW among black mothers (Table 5). A similar magnitude of effects was observed for preterm birth. While the sentiment of tweets referencing white people was not associated with birth outcomes among white mothers, the sentiment of tweets referencing racial or ethnic minority groups was associated with a greater incidence of LBW (IR 1.08, 95% CI 1.03-1.14) and preterm birth (IR 1.08, 95% CI 1.00-1.17) among non-Hispanic white mothers (Table 5).

On examining the association between negative sentiment and birth outcomes over time, there was evidence of an interaction between sentiment referencing black people and year. As a result, we present the absolute differences in the proportions and numbers of LBW and preterm births by year in Table 6 for the associations between negative tweets referencing black people and birth outcomes of black mothers, as well as the associations between tweets referencing racial or ethnic minorities and the birth outcomes of the entire population. For black mothers, the associations became stronger over time. For example, in 2015, black mothers living in states in the highest tertile for negative tweets referencing black people had a 0.65% difference in the proportion of LBW, translating to an excess of 3039 LBW babies as compared with that for mothers living in states in the lowest tertile for negative sentiment. In 2017, this increased to a difference of 1.82% or 8711 LBW babies.

**Table 4:** State-level sentiment toward racial or ethnic minorities and individual-level birth outcomes.

State-level Twitter-derived variables (tertiles for race-related tweets that are negative)		Low birth weight^a,b^, incidence ratio (95% CI) or n		Preterm birth^a,b^, incidence ratio (95% CI) or n	
**Total sample**					
	Second tertile vs first tertile (lowest)		1.08 (1.03-1.13)		1.09 (1.04-1.13)
	Third tertile		1.08 (1.04-1.13)		1.08 (1.00-1.14)
	Number		9,985,402		9,988,030
**Minorities**					
	Second tertile vs first tertile (lowest)		1.12 (1.04-1.19)		1.10 (1.05-1.15)
	Third tertile		1.13 (1.06-1.21)		1.10 (1.05-1.16)
	Number		4,920,300		4,921,577
**White people**					
	Second tertile vs first tertile (lowest)		1.07 (1.02-1.12)		1.09 (1.03-1.15)
	Third tertile		1.08 (1.03-1.14)		1.08 (1.00-1.17)
	Number		5,407,779		5,409,230

^a^Data sources for health outcomes were 2015, 2016, and 2017 natality files. Tweets were collected from June 2015 to December 2017.

^b^Adjusted log binomial models were run for each outcome separately. Models were controlled for year and state-level factors including percent non-Hispanic black people, percent Hispanic people, southern state indicator, population density, and economic disadvantage (standardized factor score summarizing the following variables [%]: unemployed, some college education, high school diploma, children in poverty, single parent household, and median household income), as well as individual-level factors including maternal age, sex, race, ethnicity, foreign birth, education, marital status, smoking, body mass index, first birth status, and prenatal care. Twitter-derived characteristics were categorized into tertiles, with the lowest tertile serving as the reference group. Cluster-adjusted errors are reported.

**Table 5:** Stratified analyses of associations between state-level sentiment and birth outcomes among subgroups.

State level sentiment toward specific groups (tertiles for tweets that are negative)		Low birth weight^a,b^, incidence ratio (95% CI) or n	Preterm birth^a,b^, incidence ratio (95% CI) or n
**Middle Eastern people and Muslims (minorities)**			
	Second tertile vs first tertile (lowest)	1.09 (1.04-1.14)	1.07 (1.03-1.12)
	Third tertile	1.07 (1.02-1.12)	1.05 (1.02-1.09)
	Number	4,920,300	4,921,577
**Black people**			
	Second tertile vs first tertile (lowest)	1.10 (1.04-1.17)	1.10 (1.06-1.16)
	Third tertile	1.08 (1.03-1.14)	1.09 (1.04-1.15)
	Number	1,413,336	1,413,938
**Hispanic people**			
	Second tertile vs first tertile (lowest)	0.96 (0.87-1.06)	0.96 (0.94-0.99)
	Third tertile	0.96 (0.89-1.04)	0.90 (0.84-0.97)
	Number	2,254,029	2,254,401
**Asian people**			
	Second tertile vs first tertile (lowest)	0.98 (0.91-1.04)	1.02 (0.97-1.07)
	Third tertile	1.03 (0.93-1.13)	1.10 (1.00-1.21)
	Number	599,580	599,769
**White people**			
	Second tertile vs first tertile (lowest)	1.01 (0.97-1.04)	1.00 (0.96-1.03)
	Third tertile	1.02 (0.97-1.07)	0.98 (0.93-1.04)
	Number	5,407,779	5,409,230

^a^Data sources for health outcomes were 2015, 2016, and 2017 natality files. Tweets were collected from June 2015 to December 2017.

^b^Adjusted log binomial models were run for each outcome separately. Models were controlled for year and state-level factors including percent non-Hispanic black people, percent Hispanic people, southern state indicator, population density, and economic disadvantage (standardized factor score summarizing the following variables [%]: unemployed, some college education, high school diploma, children in poverty, single parent household, and median household income), as well as individual-level factors including maternal age, sex, race, ethnicity, foreign birth, education, marital status, smoking, body mass index, first birth status, and prenatal care. Twitter-derived characteristics were categorized into tertiles, with the lowest tertile serving as the reference group. Cluster-adjusted errors are reported.

**Table 6:** Differences in the absolute numbers and proportions of low birth weight and preterm births between mothers living in states in the highest tertile for negative racial sentiment and mothers living in states in the lowest tertile.

Year	Low birth weight, n/N (%)		Preterm, n/N (%)	
	Total^a^	Black^b^	Total^a^	Black^b^
2015	11,712/3,444,706 (0.34)	3,039/469,659 (0.65)	14,261/3,444,783 (0.41)	3,466/470,019 (0.74)
2016	23,598/3,506,457 (0.67)	3,391/477,984 (0.71)	23,737/3,506,174 (0.68)	4,415/478,272 (0.92)
2017	10,490/3,040,622 (0.35)	8,711/479,384 (1.82)	16,827/3,037,346 (0.55)	7,060/465,674 (1.52)

^a^For the total sample, exposure is negative sentiment tweets referencing racial or ethnic minorities.

^b^For the sample of black mothers, exposure is negative sentiment tweets referencing black people.

Sensitivity analyses were conducted by modeling the ratio of negative to positive sentiments to investigate whether the findings were robust for modeling different polarities of sentiment. The findings showed a similar pattern (Multimedia Appendix 4) as compared to that for modeling negative sentiment alone, where states with a greater proportion of negative to positive tweets toward racial or ethnic minorities had a higher incidence of LBW and preterm birth.

## Discussion

This study found that negative sentiment toward racial and ethnic minorities, expressed in tweets geolocated to states, was associated with LBW and preterm birth. These adverse associations were similar for the population of all births, births in non-Hispanic white mothers, and births in racial or ethnic minorities overall. Negative tweets referencing black people were associated with adverse birth outcomes for black mothers. Similarly, negative tweets referencing Middle Eastern people were associated with poor birth outcomes among minorities. Associations were not consistently observed for negative tweets referencing non-Hispanic white or Hispanic mothers. While associations tended to be stable over the period from 2015 to 2017, for black mothers, the association between racial sentiment referencing black people and adverse birth outcomes became stronger over time.

This is among the few papers utilizing social media data to assess the racial climate in relation to health outcomes. Moreover, we did so on a national basis and accounted for individual characteristics. The results are consistent with prior work showing that the community-level racial climate is related to birth outcomes [19,20] and mortality [42] in the area. Stress has been identified as a pathway through which discrimination may impact health, and it is a known risk factor for adverse birth outcomes [43]. However, other pathways are possible, including access to resources such as education, employment, health care, and housing [3].

Previous research has provided evidence for the influence of the social context on the health of communities. Past studies have compared birth outcomes before and after a single-site immigration raid [19], the attacks on September 11, 2001 [20], and the 2016 presidential election [44] and found elevated adverse birth outcomes for minority populations following these events. One limitation of these studies is that the social context was not measured. Thus, we cannot directly evaluate whether area-level racial bias explained the association between the events and birth outcomes. Developing place-level measures of racial bias will advance the field and provide new opportunities to investigate the role of the social context in shaping health and health disparities.

Our results indicate that negative sentiment tweets referencing racial or ethnic minorities impacted the total population including non-Hispanic white people. Prior studies on racial bias and discrimination have tended to only examine the impact on racial and ethnic minorities. This study is unusual as it examined the health outcomes of the total population. A social climate that is hostile to racial and ethnic minorities might create an environment that is detrimental to all, including white people. This is consistent with prior work indicating that social cohesion promotes population health [45,46]. Animus toward racial and ethnic minorities may lead to withdrawal of support for shared resources and social policies and programs that might benefit white people and other racial and ethnic groups [47,48]. Prior work has found that living in black-segregated areas is associated with poor birth outcomes for black as well as white mothers [49,50]. There have been a few studies investigating the negative cognitive and affective impacts of racism on the perpetrators [51,52].

Although the rates of adverse birth outcomes have declined for all groups over the past century, a marked racial disparity has persisted. Similar disparities prevail for many other outcomes, including maternal mortality [53] and many adult morbidities and causes of death [54]. Interpersonal and structural racism are leading explanations for the continuing racial disparities in health, but research to confirm the causal role of racism and evaluate trends of the impact of racism on health outcomes has been hampered by the challenge of measuring racism. Our approach has important advantages in that it is easily measured and monitored, does not depend on self-reporting, is available nationally, and could likely be extended globally.

Nonetheless, the study has some limitations. The analyses did not take into account residential histories and the length of time individuals lived in their current communities. The data collected represent what people were willing to express on Twitter. Twitter users are not representative of the US population, with younger populations being over-represented on Twitter as compared with the US population [55]. However, the use of social media has been steadily increasing over time. Access to the internet and social media via cell phones has enabled people from all socioeconomic strata to engage on social media.

While the sentiment analysis represents a substantial contribution to the creation of an area-level measure of racial sentiment, there are important limitations to sentiment analysis. The sentiment analysis used the entire tweet to assess the sentiment or emotional tone of the tweet rather than focusing on just the racial terms mentioned in the tweet. Similarly, coders, who manually labeled tweets to provide training data for the machine learning algorithm, labeled the emotional tone of the tweet as a whole. Thus, it is possible that while the tone of the tweet may be negative, the race or ethnicity referenced in the tweet may not be the subject of that negativity, which was the case in many of the tweets. Additionally, the emotional tone of the tweet may display a negative sentiment, but it does not necessarily express a prejudiced statement, which was also common in our data. Our prior research indicated that prejudiced tweets can be distinct from the sentiment of the tweet [41]. For some tweets, negative sentiment also expressed negative racial attitudes or prejudiced beliefs (eg “Middle Eastern/Arabic accents piss me off more than most things.”) However, there were also negative sentiment tweets using race-related terms that did not express prejudiced beliefs. We commonly noted this with the term “nigga” (eg, “Can’t Watch The (professional basketball team) play. These Niggas Boring AF”). We also came across tweets where the sentiment was positive, but they expressed a prejudiced belief or racial or ethnic stereotype (eg, “Must have hired a Mexican cleaning crew. Bathroom got the fabuloso clean smell”). Regardless, the associations observed in our study seem to capture a signal related to the average level of racial attitudes and birth outcomes. Future work is needed to develop models to capture race-related topics as well as sentiment and to align the Twitter-based characterization of racial context to other measures of structural or interpersonal racism.

This study contributes to the nascent body of literature on place-level indicators of racial attitudes and bias. While not comprehensive, our measure of racial sentiment may represent a signal of the broader social and cultural context in which mothers reside. Data collected from Twitter may be unique as compared with what can be obtained from traditional surveys on racial attitudes or bias. Social media can represent a rich source of timely data regarding perspectives on a range of topics, including racial attitudes. This study revealed that the racial climate toward minorities may have implications for racial or ethnic minorities, as well as the entire population. The promotion of a social climate of respect, positivity, and inclusion may have beneficial health impacts for birth outcomes in the population at large.

## Appendix

Multimedia Appendix 1 Terms used in Twitter data collection.

Multimedia Appendix 2 Geographic distribution of negative sentiment tweets using race-related terms, 2015-2017.

Multimedia Appendix 3 Geographic distribution of positive sentiment tweets using race-related terms, 2015-2017.

Multimedia Appendix 4 Ratio of negative to positive sentiment toward race or ethnic minorities and individual level birth outcomes.

## Abbreviations

IR: incidence ratio
LBW: low birth weight
