# Supplementary material for: The Association Between State-Level Racial Attitudes Assessed From Twitter Data and Adverse Birth Outcomes: Observational Study
Source: JMIR Public Health Surveill. 2020 Jul 6;6(3):e17103. doi: 10.2196/17103 (PMC7381033; doi:10.2196/17103)
Supplement: Multimedia Appendix 1 [file publichealth_v6i3e17103_app1.docx]

| **Multimedia Appendix 1. Terms used in Twitter data collection** | |
| --- | --- |
| abgs | immigration |
| afghanistan | indian |
| afghanistani | indonesian |
| afghans | iranian |
| african american | iraqi |
| african americans | iroquois indian |
| african't | iroquois nation |
| africoon | iroquois tribe |
| afro caribbean | islam |
| afro-caribbean | islamic |
| aid refugees | israeli |
| alaska native | israelis |
| all lives | jamaican |
| alllifematters | jamaicans |
| alllivesmatter | jap |
| almond shaped eyes | japanese |
| american indian | jewish |
| an abg | jews |
| anglo | jig-abdul |
| apache indian | jigaboo |
| apache nation | jigarooni |
| apache tribe | jigga |
| arab | jiggabo |
| arabic | jigger |
| arabs | jihad |
| arabush | jihadi |
| aryan | jihadis |
| Aryans | jihads |
| asian | jim crow |
| asian indian | jordanian |
| asians | kafeir |
| aznbbygirl | karen people |
| bahamian | kenyan |
| bahamians | kkk |
| bamboo coon | knuckle-dragger |
| ban islam | korean |
| ban muslim | koreans |
| ban on mulsims | kuffar |
| bangalees | kyke |
| bangladeshi | laotian |
| banislam | latin american |
| banjo lip | latina |
| banmuslim | latinas |
| banonmulsims | latino |
| bantu | latinos |
| beaner | lebanese |
| beaner shnitzel | liberian |
| beanershnitzel | light skin |
| bengalis | ling ling |
| bhutanese | little hiroshima |
| biscuit lip | malayali |
| bix nood | malaysian |
| black boy | mexcrement |
| black boys | mexican |
| black female | mexicans |
| black girl | mexican't |
| black girls | mexico border |
| black history | mexicoborder |
| black lives | mexicoon |
| black male | mexihos |
| black man | middle eastern |
| black men | minorities |
| black twitter | mongolian |
| black woman | mongolians |
| black women | moroccan |
| blacklivesmatter | moroccans |
| blacklivesmatter | moulie |
| blacks | mozambican |
| blacktwitter | mud people |
| blktwiter | mudshark |
| blktwitter | muslim |
| blue eye devil | muslimban |
| blue lives | muslims |
| bohunk | muzrat |
| bootlip | muzzie |
| border bandit | n word |
| border control | native american |
| border fence | native americans |
| border hopper | native hawaiian |
| border jumper | navajo |
| border nigger | negro |
| border security | negroes |
| border surveillance | negros |
| border wall | nepalese |
| bounty bar | new mexico border |
| bow bender | nigerian |
| brazilians | nigerians |
| brown people | nigette |
| buckra | nigga |
| buddhahead | niggah |
| buffalo jockey | niggas |
| buffie | nigger |
| build a wall | niggers |
| buildawall | nigglet |
| bumper lip | nigglets |
| burmese | niggress |
| burnt cracker | niglet |
| burrhead | nig-ngo |
| burundi | noodle nigger |
| bush-boogie | north korean |
| bushnigger | n-word |
| cairo coon | oriental |
| cambodian | orientals |
| cambodians | our country back |
| camel cowboy | ourcountryback |
| camel fucker | pacific islander |
| camel jacker | paki |
| camelfucker | pakistani |
| camel-fucker | palestinian |
| cameljacker | panamanian |
| camel-jacker | paraguayan |
| carpet pilot | pashtun |
| carpetpilot | pegida |
| carribean people | people of color |
| caublasian | peruvian |
| caucasian | pickaninny |
| caucasians | pisslam |
| central american | poc |
| chain dragger | polynesian |
| chamorro | porch monkey |
| cherokee indian | prairie nigger |
| cherokee nation | pueblo indians |
| cherokee tribe | pueblo nation |
| cherry nigger | pueblo tribe |
| chexican | puerto rican |
| chicano | puerto ricans |
| chicanos | qtip head |
| chiegro | qwhite |
| chinaman | race traitor |
| chinese | racism |
| ching chong | racist |
| ching-chong | racists |
| chink | rag head |
| chinks | raghead |
| chippewa indian | rapefugee |
| chippewa nation | red nigger |
| chippewa tribe | refugee |
| choctaw indian | refugees |
| choctaw nation | resettlement |
| choctaw tribe | rice burner |
| chonky | rice nigger |
| clit chopper | rice rocket |
| clit-chopper | rice-nigger |
| clitless | river nigger |
| clit-swiper | rivernigger |
| coconut nigger | rug pilot |
| colombian | rug rider |
| columbians | rugpilot |
| congo lip | rwandan people |
| congolese | salvadoreans |
| coolie | samoan |
| coon | sanctuary cities |
| coonass | sanctuary city |
| coon-ass | sanctuarycities |
| coontang | sanctuarycity |
| costa rican | sand flea |
| cracker jap | sand monkey |
| cuban | sand moolie |
| cubans | sand nigger |
| cunt-eyed | sand rat |
| dampback | sandflea |
| dark skin | sandmonkey |
| darkey | sandmoolie |
| darkie | sandnigger |
| darky | sandrat |
| deport | second generation + immigrant |
| deportation | secure our border |
| deported | secureourborder |
| deporting | shiptar |
| deports | sideways cooter |
| derka derka | sideways pussy |
| derkaderka | sioux indian |
| diaper head | sioux nation |
| diaperhead | sioux tribe |
| diaper-head | sjw |
| dink | Skinhead |
| dog muncher | slanted eye |
| dog-muncher | slant-eye |
| dominican | slave |
| dominicans | slavery |
| dothead | slaves |
| dune coon | slopehead |
| dune nigger | slurpee nigger |
| dunecoon | slurpeenigger |
| dunenigger | somali |
| durka durka | somalian |
| durka-durka | south african |
| east asian | south american |
| ecuadorian | south asian |
| egyptian | spearchucker |
| egyptians | spic |
| end sanctuary | spick |
| ethiopian | spig |
| ethiopians | spigotty |
| fence fairy | spik |
| fence hopper | squaw |
| fence-hopper | squinty |
| fesskin | sudanese |
| field nigger | sun goblin |
| filipino | syria |
| filipinos | syrian |
| fingernail rancher | syrianrefugee |
| first generation + immigrant | syrians |
| fob | taco nigger |
| freddie gray | taiwanese |
| freddiegray | Tamir + Rice |
| fresh off | tamirrice |
| fuckmuslims | tanzanian |
| gable | tar baby |
| ghanaian | tar-baby |
| ghetto | teepee creeper |
| go back where | tee-pee creeper |
| gobackwhere | thai |
| golliwog | thais |
| gook | thin eyed |
| gookaniese | thin-eyed |
| gookemon | tibetan |
| gooky | timber nigger |
| Gringo | timbernigger |
| groid | tomahawk chucker |
| guamanian | tomahawk-chucker |
| guatemalans | tomahonky |
| guido | towel head |
| gyppo | towelhead |
| haitian | towel-head |
| haitians | Trailer Trash |
| half breed | undocumented |
| half cast | unhcr |
| half-breed | vietnamese |
| half-cast | we welcome refugees |
| hambaya | welcome refugee |
| hatchet-packer | welcomerefugee |
| hebe | wetback |
| heeb | wetblack |
| help refugees | whacky iraqi |
| hijab | whigger |
| hijabi | white boy |
| hijabs | white boys |
| Hillbilly | White Devil |
| hilljack | white girl |
| hillwilliam | white girls |
| hindu | white man |
| hindus | white men |
| hispandex | white people |
| hispanic | white trash |
| hispanics | white woman |
| honkey | white women |
| honkeys | whitegenocide |
| honky | whites |
| honkys | whitetrash |
| hotep | whitey |
| hymie | whiteys |
| ikey | wigga |
| illegal alien | wigger |
| illegal aliens | wog |
| illegal immigrant | wypipo |
| illegal immigrants | zambian |
| immigrant | zimbabwean |
| immigrants | zipperhead |
