# Supplementary material for: The Association Between State-Level Racial Attitudes Assessed From Twitter Data and Adverse Birth Outcomes: Observational Study
Source: JMIR Public Health Surveill. 2020 Jul 6;6(3):e17103. doi: 10.2196/17103 (PMC7381033; doi:10.2196/17103)
Supplement: Multimedia Appendix 3 [file publichealth_v6i3e17103_app3.docx]

Multimedia Appendix 3. Geographic distribution of positive sentiment tweets using race-related terms, 2015-2017


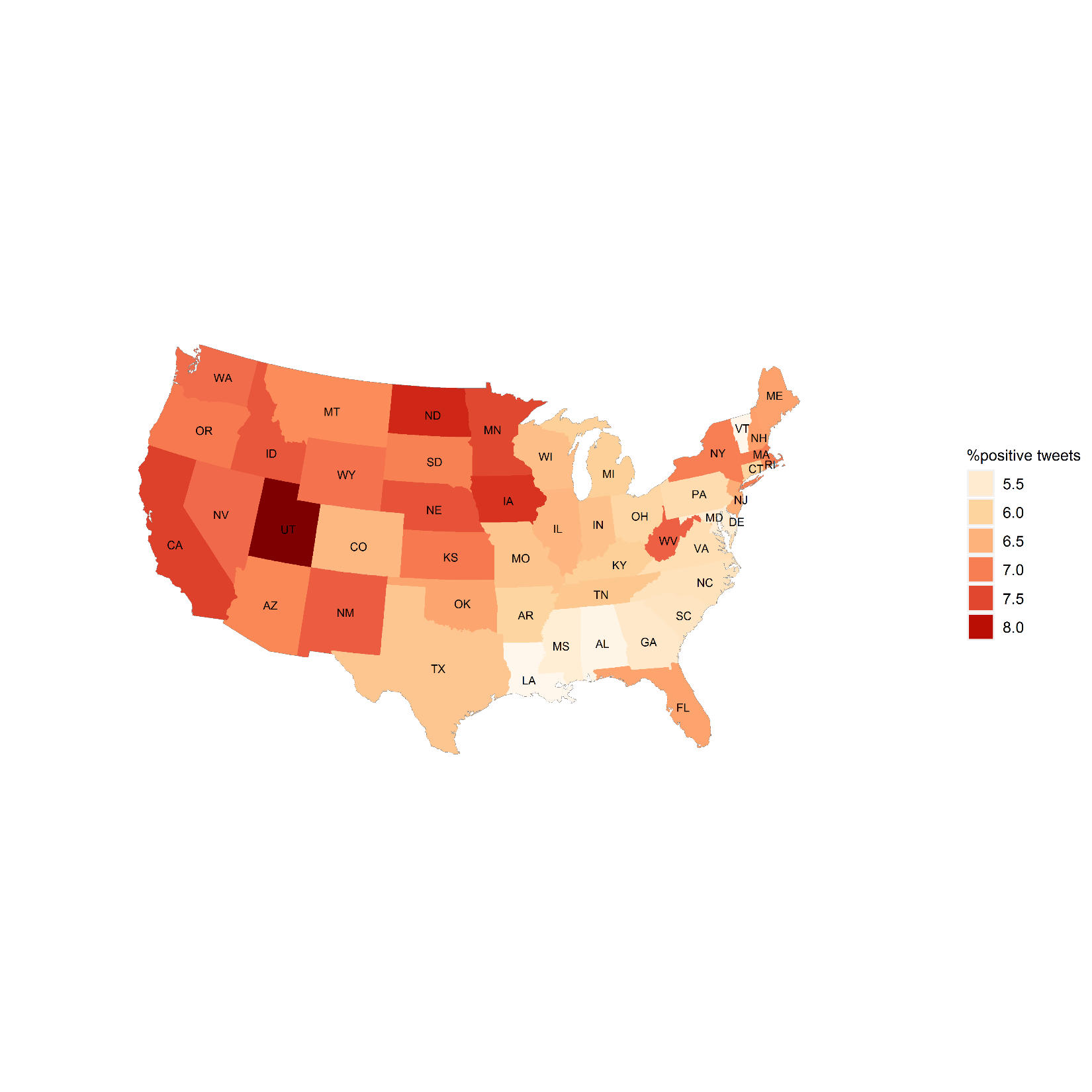


Mapping negative Twitter-derived sentiment. State level summaries of percent of tweets referencing racial/ethnic minorities that are negative. Choropleth maps were created using R package ggplot2 and package map.
