# Supplementary material for: The Association Between State-Level Racial Attitudes Assessed From Twitter Data and Adverse Birth Outcomes: Observational Study
Source: JMIR Public Health Surveill. 2020 Jul 6;6(3):e17103. doi: 10.2196/17103 (PMC7381033; doi:10.2196/17103)
Supplement: Multimedia Appendix 4 [file publichealth_v6i3e17103_app4.docx]

| Multimedia Appendix 4. Ratio of negative to positive sentiment towards race/ethnic minorities and individual level birth outcomes | | |
| --- | --- | --- |
|  | Low Birth Weight | Preterm birth |
| *State level Twitter-derived variables* | Prevalence Ratio  (95% CI) | Prevalence Ratio  (95% CI) |
| Ratio of negative to positive race-related tweets | | |
| **Total Sample** |  |  |
| 2nd tertile vs 1st (lowest) | 1.05 (1.02, 1.09) | 1.05 (1.01, 1.09) |
| 3rd tertile | 1.06 (1.02, 1.11) | 1.05 (0.99, 1.12) |
| N | 10,024, 081 | 10,026,728 |
| **Among Minorities** |  |  |
| 2nd tertile vs 1st (lowest) | 1.12 (1.06, 1.19) | 1.09 (1.04, 1.14) |
| 3rd tertile | 1.15 (1.08, 1.23) | 1.10 (1.04, 1.17) |
| N | 4,920,300 | 4,921,577 |
| **Among Whites** |  |  |
| 2nd tertile vs 1st (lowest) | 1.05 (1.00, 1.10) | 1.05 (1.00, 1.10) |
| 3rd tertile | 1.06 (1.00, 1.13) | 1.05 (0.97, 1.14) |
| N | 5,407,779 | 5,409,230 |
| **Specific Subgroup Associations** | | |
| **Middle Easterners (among Minorities)** | | |
| *Ratio of negative to positive tweets referencing Middle Easterners including Muslims* | | |
| 2nd tertile vs 1st (lowest) | 1.04 (0.99, 1.08) | 1.03 (1.00, 1.07) |
| 3rd tertile | 1.03 (0.98, 1.09) | 1.02 (0.98, 1.06) |
| N | 4,920,300 | 4,921,577 |
| **Among Blacks** |  |  |
| *Ratio of negative to positive tweets referencing Blacks* | | |
| 2nd tertile vs 1st (lowest) | 1.07 (1.03, 1.11) | 1.08 (1.03, 1.13) |
| 3rd tertile | 1.03 (0.98, 1.08) | 1.04 (0.99, 1.10) |
| N | 1,413,336 | 1,413,938 |
| **Among Hispanics** |  |  |
| *Ratio of negative to positive race-related tweets referencing Hispanics* | | |
| 2nd tertile vs 1st (lowest) | 1.07 (1.03, 1.11) | 1.08 (1.03, 1.13) |
| 3rd tertile | 1.03 (0.98, 1.08) | 1.04 (0.99, 1.10) |
| N | 2,254,029 | 2,254,401 |
| **Among Asians** |  |  |
| *Ratio of negative to positive tweets referencing Asians* | | |
| 2nd tertile vs 1st (lowest) | 1.00 (0.93, 1.07) | 1.03 (0.98, 1.08) |
| 3rd tertile | 1.06 (0.98, 1.16) | 1.07 (0.94, 1.22) |
| N | 599,580 | 599,769 |
| **Among Whites** |  |  |
| *Ratio of negative to positive tweets referencing Whites* | | |
| 2nd tertile vs 1st (lowest) | 0.97 (0.92, 1.03) | 0.99 (0.96, 1.03) |
| 3rd tertile | 0.95 (0.90, 1.00) | 0.94 (0.89, 0.99) |
| N | 5,407,779 | 5,409,230 |
| ^a^Data source for health outcome: 2015, 2016, and 2017 Natality Files. Tweets collected from June 2015 - December 2017 | | |
| ^b^Adjusted log binomial models were run for each outcome separately. Models controlled for year and state-level % non-Hispanic Black, % Hispanic, southern state indicator, population density and economic disadvantage (standardized factor score summarizing the following four variables: percent unemployed; percent with some college, percent with high school diploma, percent children in poverty, percent single parent households, and percent median household income) as well as individual level maternal age, sex, race, ethnicity, foreign birth, education, marital status, smoking, body mass index, first birth status, and prenatal care. Twitter-derived characteristics were categorized into tertiles, with the lowest tertile serving as the referent group. Cluster adjusted errors reported. | | |
